# Natural Bicarbonate Water Might Enhance Nitrogen Balance and Lipid Metabolism and Improve Calcium Balance: A Full Quantitative Targeted Metabolomics Study in Rats

**DOI:** 10.3390/nu17111875

**Published:** 2025-05-30

**Authors:** Jiaohua Luo, Jia Wang, Zhiqun Qiu, Hui Zeng, Yao Tan, Yujing Huang, Weiqun Shu

**Affiliations:** 1 Department of Environmental Hygiene, College of Preventive Medicine, Army Medical University, Chongqing 400038, China; ljh978@tmmu.edu.cn (J.L.); qiuzhiqun@tmmu.edu.cn (Z.Q.); zenghui@tmmu.edu.cn (H.Z.); xiaoyue7122@tmmu.edu.cn (Y.T.); 2 Department of Medical English, College of Basic Medicine, Army Medical University, Chongqing 400038, China; awcm@tmmu.edu.cn

**Keywords:** natural bicarbonate water, purified water, metabolomics analysis

## Abstract

**Background/Objectives**: Drinking natural bicarbonate water (NBW) has been associated with decreased bone resorption, improved lipid profile, and reduced cardiovascular risk. However, the specific molecular mechanisms underlying these effects remain unclear. **Methods**: Twenty 10-month-old female Sprague Dawley rats were randomly allocated to two experimental groups; one received purified water (PW) and the other was administered NBW over a three-month intervention period. The liver’s metabolic properties were analyzed using a comprehensive quantitative targeted metabolomics technique. **Results**: Sixty-nine differential metabolites (67 upregulated and 2 downregulated) were detected in the NBW group compared to the PW group. These metabolites included 34 amino acids, 11 carbohydrates, 7 fatty acids, 7 short-chain fatty acids (SCFAs), and 10 other biomolecules. Furthermore, 10 metabolic pathways exhibited significant alterations: aminoacyl-tRNA biosynthesis; alanine-aspartate-glutamate metabolism; nitrogen-butanoate metabolism; histidine-phenylalanine metabolism; arginine-proline metabolism; glycine-serine-threonine metabolism; valine-leucine-isoleucine biosynthesis; and phenylalanine-tyrosine-tryptophan biosynthesis. The NBW group demonstrated a statistical tendency toward lower urinary calcium/creatinine ratio compared to the PW group. **Conclusions**: These findings suggest that the consumption of NBW may induce positive nitrogen balance, enhance the level of certain polyunsaturated fatty acids and SCFAs, and improve calcium balance. Such metabolic alterations could potentially explain the beneficial effects of NBW.

## 1. Introduction

Water accounts for about 60% of total body mass in Homo sapiens, serving critical roles in homeostatic maintenance through four principal mechanisms: thermoregulation; the transport of nutrients, oxygen, and bioactive molecules; the clearance of metabolic wastes; and hydration essential for articular/tissue biomechanics [1]. A daily intake of 1.5 to 2 L of water is essential, as adequate hydration is vital for maintaining water equilibrium in the body [1]. Beyond maintaining hydration, water consumption delivers essential minerals that modulate physiological well-being through dual mechanisms: direct biochemical interactions; and the indirect regulation of nutrient assimilation and metabolic pathways [2]. Many individuals opt for mineral water to fulfill their daily hydration needs; the intake of mineral water has witnessed a dramatic surge since the late 20th century [3]. Natural bicarbonate water (NBW) is characterized by its cold and alkaline nature, along with its low mineral content [1]. As the primary alkaline reserves in human plasma, bicarbonate is instrumental in regulating acid–base balance. The intake of NBW can elevate the dietary alkali load, thereby reducing net acid excretion by counteracting the dietary acid load and mitigating the risks associated with chronic metabolic acidosis [4,5]. The consumption of NBW has been associated with decreased levels of parathyroid hormone and reduced bone resorption in healthy young women [6,7], as well as inhibited serum aldosterone levels in healthy participants [8,9]. Additionally, it has been shown to decrease systolic blood pressure [10,11], improve lipid profiles, and reduce cardiovascular risk in moderately hypercholesterolemic young men and women [10]. Furthermore, the consumption of NBW has been reported to enhance glycemic control, prevent or ameliorate type 2 diabetes in humans [12], and alleviate dyspeptic symptoms [13].

Although the consumption of NBW is known to confer health benefits, the precise mechanisms mediating these bioeffects remain unclear. Metabolomics employs advanced analytical techniques and computational methods to analyze global biochemical alterations within biological systems, thereby providing comprehensive metabolic phenotyping and biomarkers [14]. Metabolites, being the final downstream products of gene transcription and protein translation, serve as a crucial link between genotypes, the environment, and phenotypes. They provide a distinctive metabolic profile and real-time health assessment, revealing critical data on the downstream metabolites linked to diverse biochemical pathways [15]. Mass spectrometry-driven metabolomics facilitates the high-throughput identification and quantitative analysis of diverse metabolite pools, covering thousands of molecular species in a single assay [16]. The liver functions as the mammalian body’s metabolic center, orchestrating amino acid/fatty acid/carbohydrate metabolism, bile acid and hormone synthesis, lipoprotein biogenesis, and detoxification processes. Relative to young adult rats, perimenopausal rats exhibit significantly reduced serum estradiol levels, diminished bone mineral density, and decreased bone mineral content, which demonstrates elevated susceptibility to osteoporosis and cardiovascular disorders [17]. Therefore, this study aims to investigate the mechanisms of the beneficial effects in perimenopausal rats exerted by NBW consumption. We conducted a comparative analysis of hepatic metabolic profiles between 10-month-old female rats receiving purified water (PW) and those receiving NBW.

## 2. Materials and Methods

### 2.1. Drinking Water and Food

This experiment utilized two types of water: bottled PW and bottled NBW, both obtained from a supermarket. The water quality was examined according to GB/T 5750-2006 Standard [18] examination methods and is presented in Table 1. The rat food was purchased from the Experimental Animal Center of Army Medical University (Certification No. SCX-2007-018), formulated in full compliance with both of China’s GB14924-2010 nutritional standards [19]. Macronutrient composition and contaminant thresholds are cataloged in Supplementary Table S1.

### 2.2. Animals and Experiment Design

All animal experiments were conducted under protocols approved by the Army Medical University Institutional Animal Care and Use Committee (Approval No: AMUWEC20198024), with operations performed by licensed personnel in full compliance with NIH standards and ARRIVE guidelines. Specific-pathogen-free (SPF) female Sprague Dawley rats (age: 10 months; weight: 360–380 g) were obtained from the university’s certified Animal Experimental Center (Production License SCXK-2017-0002) and acclimatized for 7 days in controlled SPF conditions (temperature: 25 ± 1 °C, humidity: 50 ± 5%, 12 h/12 h light/dark cycle).

The rats were randomly divided into two groups (*n* = 10/group) receiving either PW or NBW ad libitum for three months. Daily monitoring included food/water consumption measurements, with weekly body weight recordings. All procedures strictly maintained free access to nutrition while preventing pathogen exposure.

Following the 3-month experimental period, rats were individually housed in metabolic cages for 24 h urine collection. Then, the rats underwent terminal blood collection through cardiac puncture under deep anesthesia induced by intraperitoneal sodium pentobarbital. Serum was separated via centrifugation at 3000× *g* for 15 min at 4 °C and aliquoted for storage. The median liver lobes were immediately excised, snap-frozen in liquid nitrogen within 30 s of extraction, and subsequently transferred to cryogenic storage at −80 °C for long-term preservation.

### 2.3. Biological Sample Analysis

Serum and urinary biochemical profiling were conducted using a Beckman Coulter AU5800 clinical chemistry analyzer (Brea, CA, USA). Urinalysis quantified electrolyte concentrations (K^+^, Na^+^, Ca^2+^, and Mg^2+^) and other indicators levels. Serum assays encompassed mineral concentration, lipid profile, hepatic function markers, and renal function parameters.

### 2.4. Targeted Metabolic Profiling

Liver metabolic characterization was performed using the Q300 Quantitative Metabolite Assay Kit (Metabo-Profile, Shanghai, China) with ten biological replicates per experimental group (*n* = 10 rats/group). The analysis covered six major metabolic clusters: carbohydrates; amino acids; lipid species; organic acids; carnitines; and short-chain fatty acids (SCFAs). Tissue processing followed established derivatization protocols with critical adaptations [20] (refer to the Supplementary Materials and Methods Section for detailed protocols). Metabolite separation was achieved using an ACQUITY UPLC H-Class system coupled to a Xevo TQ-S tandem quadrupole mass spectrometer (Waters Corp., Milford, MA, USA), with chromatographic conditions detailed in the Supplementary Materials and Methods Section. Instrument performance optimization and routine maintenance were conducted weekly. A comprehensive quality assurance system was implemented as follows: (1) internal standards for batch-specific normalization; (2) pooled QC samples (equal-volume aliquots from all specimens) analyzed at 14-sample intervals; and (3) process blanks (extraction solvent only) and solvent blanks (LC-MS grade water) across batches.

### 2.5. Statistics

The UPLC-MS/MS raw data were analyzed using MassLynx software (v4.1, Waters Corp., Milford, MA, USA) for metabolite peak integration, calibration and quantitation. The statistical analysis was performed on the iMAP platform (v1.0, Metabo-Profile, Shanghai, China), which included both univariate and multivariate approaches. For univariate analysis, Student’s *t*-test, Mann–Whitney U-test and fold change (FC) analysis were applied with a significance threshold of *p* < 0.05 to identify differential metabolites. The multivariate analysis comprised principal component analysis (PCA) and orthogonal partial least squares discriminant analysis (OPLS-DA), where metabolites with variable importance in projection (VIP) scores > 1 were considered differential. The overlapping differential metabolites from both statistical approaches were visualized using Venn diagrams. Volcano plots were generated by combining fold change and *p*-values to highlight significant metabolite differences, with arrows (↓ for decrease, ↑ for increase) indicating the direction of changes in the NBW group. Finally, pathway enrichment analysis was performed to reveal significantly altered metabolic pathways.

Routine serum and urinary parameters were expressed as mean ± standard deviation (SD). Data analysis was performed using SPSS software (version 20.0; IBM Corp., Armonk, NY, USA). Normality of data distribution was first verified via the one-sample Kolmogorov–Smirnov test. Based on the normality results, intergroup differences were assessed using either Student’s *t*-test (for normally distributed data) or the Mann–Whitney U test (for non-normally distributed data). A threshold of *p* < 0.05 was applied to determine statistical significance.

## 3. Results

### 3.1. Water Quality Analysis

NBW demonstrated significantly elevated concentrations of total dissolved solids, total hardness, bicarbonate, calcium, magnesium, potassium, sodium, boron, and silicic acid compared to PW. Furthermore, NBW exhibited a higher pH value and a lower potential renal acid load (PRAL) value than PW (Table 1).

### 3.2. Bodyweight, Food and Water Intake

Throughout the study, both groups of rats exhibited comparable physical and behavioral profiles, with no statistically significant differences detected during monitoring. Quantitative assessments confirmed analogous patterns in daily food intake, water consumption, and body weight measurements between the groups over the experimental duration (Figure S1).

### 3.3. Serum and Urinary Biochemical Analysis

The comparative analysis revealed comparable serum and urinary mineral profiles between the groups (Table 2), although the NBW group demonstrated a trend toward a lower urinary calcium-to-creatinine ratio relative to PW controls (*p* = 0.08). Comprehensive biochemical evaluations further indicated equivalent ranges for serum lipids, protein fractions, hepatic enzymes, and renal function markers across both groups (Table 3).

### 3.4. Metabolic Profiling of Liver

**Figure 1:**
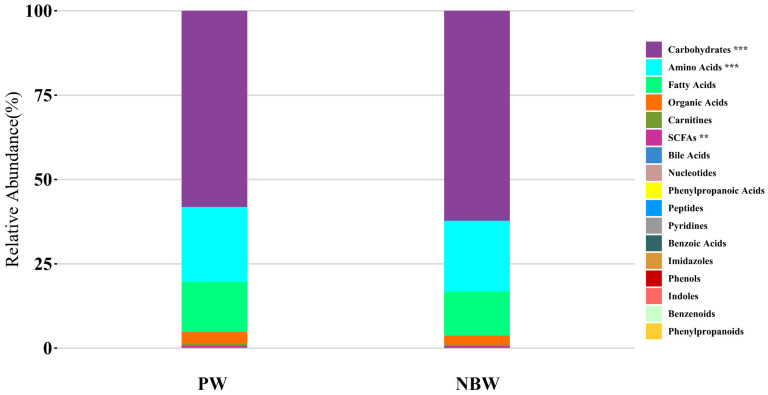
Proportional distribution of metabolite classes. **, *p* < 0.01; ***, *p* < 0.001. PW stands for the purified water, and NBW stands for the natural bicarbonate water. *n* = 10 rats/group.

Hepatic metabolite profiling was performed on rat liver specimens via ultra-performance liquid chromatography–tandem mass spectrometry (UPLC-MS/MS) analysis, revealing 209 identified metabolites classified into 17 categories. The compositional distribution was dominated by carbohydrates (60.58%), followed by amino acids (21.5%), fatty acids (13.79%), organic acids (2.94%), with minor constituents (1.19%) (Figure 1). The comparative analysis demonstrated statistically significant intergroup differences in carbohydrates (PW: 58.11% vs. NBW: 62.20%), amino acids (22.24% vs. 21.01%), and SCFAs (0.43% vs. 0.41%).

**Figure 2:**
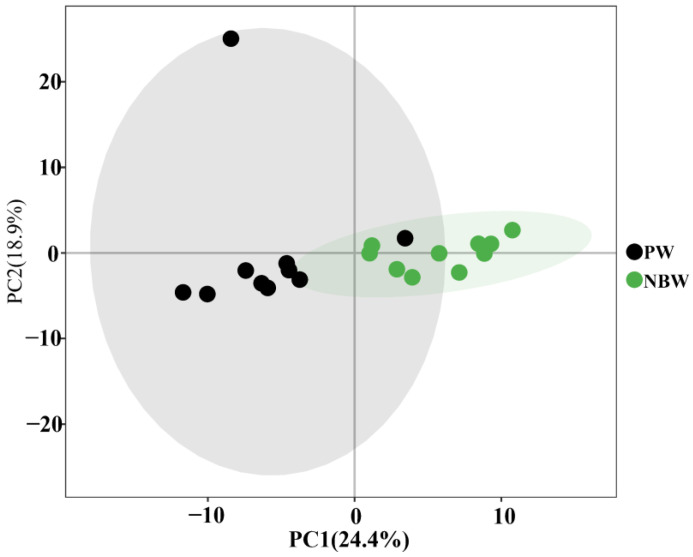
PCA 2D score plot. PW stands for the purified water and NBW stands for the natural bicarbonate water. *n* = 10 rats/group.

To characterize hepatic metabolic variations, we performed multivariate statistical analyses incorporating both unsupervised principal component analysis (PCA) and supervised orthogonal partial least squares-discriminant analysis (OPLS-DA). The PCA score plot (Figure 2) demonstrated clear intergroup differences, where: (1) each data point corresponded to a single rat liver specimen; (2) distinct clustering patterns were observed between experimental groups; (3) NBW and PW groups showed pronounced spatial segregation; and (4) the first two principal components (PC1 and PC2) collectively explained 43.3% of the total metabolic variance.

**Figure 3:**
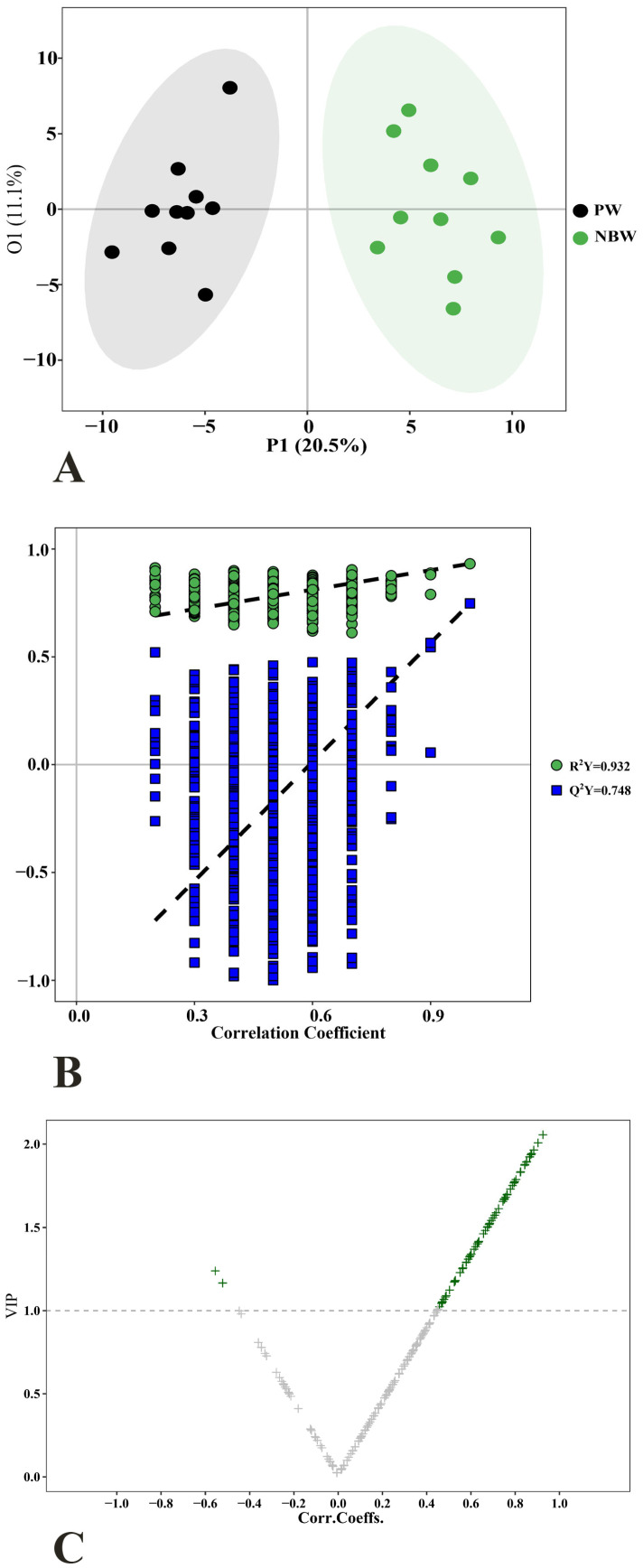
(**A**) OPLS-DA 2D score plot; (**B**) permutation plot; and (**C**) volcano plot visualizing differential metabolites in the OPLS-DA model. Green: differential metabolites (VIP > 1). PW stands for the purified water and NBW stands for the natural bicarbonate water. *n* = 10 rats/group.

The OPLS-DA score plot (Figure 3A) demonstrated clear intergroup differences, with distinct clustering patterns between groups. To ensure model robustness, we conducted a 1000-permutation validation test, which yielded parameters (R^2^Y = 0.932, Q^2^Y = 0.748) and a negative Q^2^ regression intercept (Figure 3B), collectively confirming the absence of overfitting. Metabolite discriminatory power was quantitatively assessed using VIP scores, while subsequent volcano plot analysis identified 72 significantly altered metabolites (VIP > 1) in the NBW group (Figure 3C).

**Figure 4:**
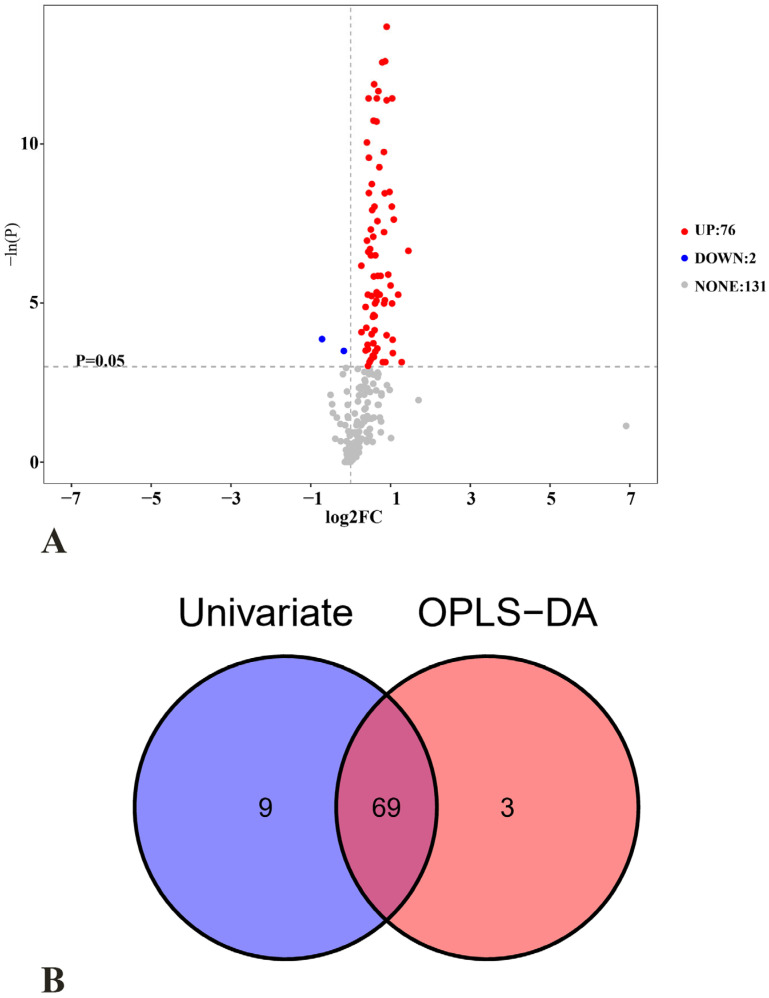
(**A**) Volcano plot of univariate statistics: differential metabolite selection threshold (*p* < 0.05, |log_2_FC| > 0); and (**B**) Venn diagram of differential metabolites identified by multivariate and univariate analyses. PW stands for purified water and NBW stands for natural bicarbonate water. *n* = 10 rats/group.

The FC and corresponding *p*-values of individual metabolites were visualized in the volcano plot of univariate analysis (Figure 4A). Differential metabolites were defined using dual criteria: *p* < 0.05 and |log_2_FC| > 0. The comparative analysis between the NBW and PW groups revealed 78 differential metabolites, with 76 demonstrating significant upregulation and 2 exhibiting downregulation.

Integration of the multivariate (OPLS-DA) and univariate analytical approaches identified candidate biomarkers. Venn diagram intersection analysis delineated 69 overlapping differential metabolites (Figure 4B), categorized as follows: 34 amino acids; 11 carbohydrates; 7 fatty acids; 7 short-chain fatty acids (SCFAs); and 10 additional biomolecules.

**Figure 5:**
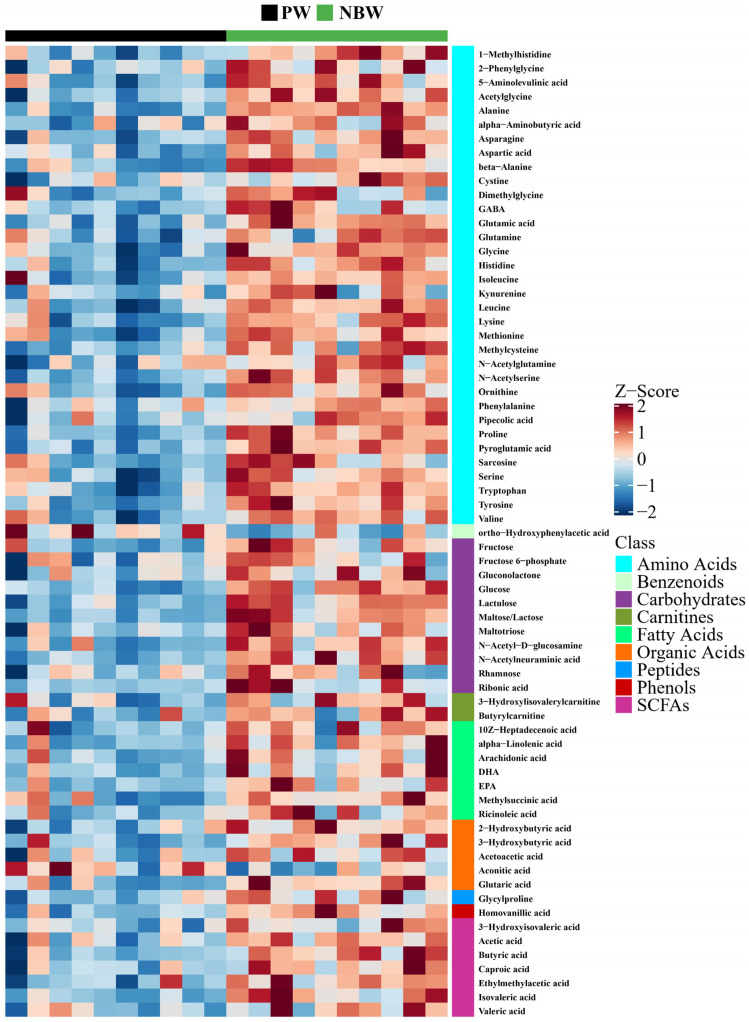
Z score plot for potential biomarkers. PW stands for purified water and NBW stands for natural bicarbonate water. *n* = 10 rats/group.

Relative to the PW group, the NBW group exhibited decreased concentrations of aconitic acid and ortho-hydroxyphenylacetic acid, whereas the remaining 67 metabolites showed significant upregulation (Figure 5 and Table 4).

**Figure 6:**
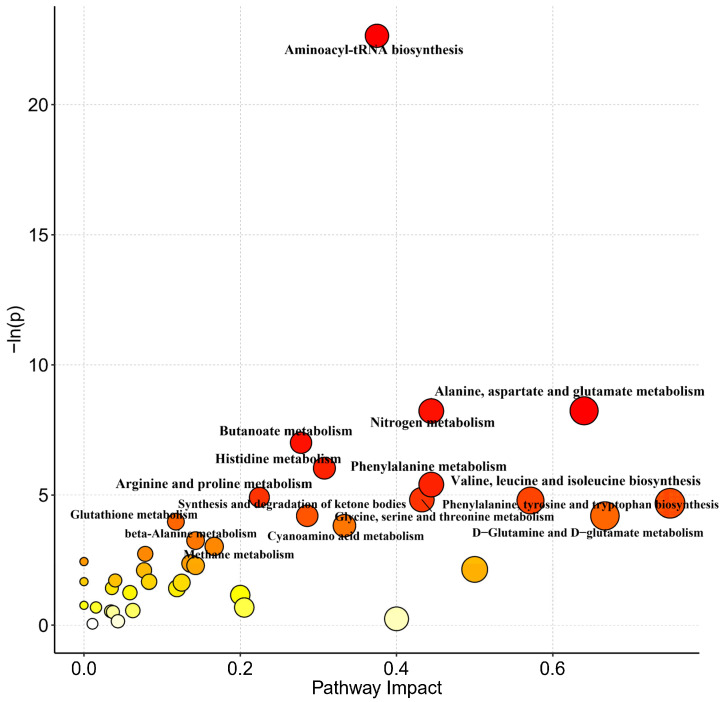
Bubble plot of pathway impact using the RNO set. The color change (yellow to red) of the circle is positively correlated with the negative logarithm of the *P*-value of pathway changes. The larger circle size represents the larger value of pathway impact. PW stands for purified water and NBW stands for natural bicarbonate water. *n* = 10 rats/group.

Metabolic pathway analysis of the 69 identified metabolites (RNO set) revealed 10 significantly altered pathways (*p* < 0.05, FDR < 0.1), including: aminoacyl-tRNA biosynthesis; alanine-aspartate-glutamate metabolism; nitrogen metabolism; butanoate metabolism; histidine metabolism; phenylalanine metabolism; arginine-proline metabolism, glycine-serine-threonine metabolism; valine-leucine-isoleucine biosynthesis; and phenylalanine-tyrosine-tryptophan biosynthesis (Figure 6 and Table 5).

## 4. Discussion

This study investigated hepatic metabolic alterations in 10-month-old female rats following a three-month administration of PW and NBW. In the NBW group, 69 differential metabolites were detected, comprising 34 amino acids, 11 carbohydrates, 7 fatty acids, 7 SCFAs, and 10 other biomolecules. Notably, 67 metabolites exhibited significant upregulation, whereas 2 were downregulated. Pathway enrichment analysis further revealed significant changes in 10 key metabolic pathways.

### 4.1. Calcium and Sodium Balance

The NBW was characterized by a higher concentration of bicarbonate and sodium. With a total hardness of 12.51 mg/L CaCO_3_, the NBW qualifies as soft water. A three-month intervention revealed no significant alterations in serum sodium levels or urinary sodium-to-creatinine ratios between the two groups, suggesting that NBW consumption does not elevate the systemic sodium burden. Notably, sodium bicarbonate-enriched water has demonstrated efficacy in lowering mean arterial pressure among normotensive elderly populations [11]. Although sodium chloride is positively associated with hypertension, recent evidence implicates chloride ions as the principal mediator of blood pressure elevation and renin–angiotensin–aldosterone system activation [21]. These findings collectively support NBW’s potential role in blood pressure modulation.

The NBW group demonstrated a statistical tendency toward a lower urinary calcium-to-creatinine ratio compared to the PW group, aligning with previous reports on the calcium-sparing effects of bicarbonate-rich water [11]. Specifically, Schorr et al. reported lower urinary calcium excretion in normotensive elderly subjects consuming bicarbonate water, while Schoppen et al. observed reduced calcium loss in postmenopausal women versus controls [22]. These consistent findings across demographic groups suggest that NBW’s high bicarbonate content may enhance renal calcium retention.

Extensive research shows that elevated sodium intake promotes urinary calcium excretion, potentially leading to negative calcium balance [23,24]. Paradoxically, our study observed reduced urinary calcium excretion following sodium bicarbonate-rich water consumption, implying that bicarbonate ions may counteract sodium-induced calcinuria. This phenomenon aligns with bone’s physiological role as a pH buffer: during metabolic acidosis, skeletal calcium mobilizes to neutralize acidity, compromising bone mineral density [25]. Bicarbonate serves as a critical acid–base regulator. Mineral waters containing bicarbonate enhance systemic alkalinity, decreasing net acid excretion and offsetting dietary acid load [4,5]. Population studies confirm these benefits, showing that alkaline water consumption improved spinal bone density in osteoporotic postmenopausal women through reduced calcinuria [26] and elevated serum ionized calcium and urine pH while suppressing parathyroid hormone and bone resorption markers in healthy postmenopausal women [27].

Notably, the NBW contained 0.97 mg/L boron—a bioactive micronutrient that modulates calcium–magnesium metabolism [28,29]. Boron supplementation demonstrates dual benefits, reducing urinary calcium/magnesium losses [30] while enhancing calcium absorption efficiency [31]. Since hypercalciuria predisposes to calcium oxalate nephrolithiasis [32], NBW’s combined bicarbonate–boron action may simultaneously improve calcium retention (reducing osteoporosis risk) and lower the incidence of kidney stones based on urinary calcium correlations.

### 4.2. Amino Acids and Protein Synthesis

Amino acids serve as fundamental building blocks for protein synthesis, forming polypeptide chains through peptide bond linkages. Both plant and animal proteins comprise the same set of approximately 20 standard amino acids, with their unique sequences determining protein specificity [33]. Amino acids are categorized as essential, non-essential, or conditionally essential, based on biosynthetic capacity. Beyond protein construction, amino acids function as critical precursors for numerous physiologically important peptides and low molecular-weight compounds [34]. Our metabolomic analysis revealed 69 differential hepatic metabolites in NBW-treated rats. Those metabolites comprised 34 amino acids, 11 carbohydrates, 7 fatty acids, 7 short-chain fatty acids, and 10 other biomolecules. Notably, all 34 detected amino acids showed elevated concentrations in the NBW group compared to the PW controls, including 8 essential amino acids (lysine, histidine, phenylalanine, valine, methionine, isoleucine, leucine, and tryptophan) that mammals cannot synthesize de novo [33]. Crucially, experimental conditions were rigorously controlled: all animals received identical food ad libitum with no intergroup differences. Hepatic and renal function markers also remained comparable between groups, excluding organ dysfunction as a confounding factor. These findings strongly suggested that NBW consumption directly modulates hepatic amino acid metabolism, potentially through enhanced absorption or metabolic pathway regulation.

The observed elevation in hepatic amino acid levels may be attributed to two synergistic mechanisms. Primarily, NBW consumption appears to enhance intestinal amino acid absorption. Dietary proteins undergo proteolytic cleavage into absorbable amino acids, which are subsequently transported across enterocytes via sodium-dependent neutral amino acid transporters (SLC6A15-SLC6A20) [35]. The elevated sodium content in NBW likely potentiates this absorptive process through sodium-dependent transporter activation. Secondarily, NBW administration stimulated hepatic non-essential amino acid synthesis. Our metabolomic data revealed significant increases in 11 carbohydrate metabolites, particularly glucose, N-acetylneuraminic acid, and lactose derivatives. These substrates fuel glycolytic and TCA cycle pathways, generating α-ketoacid precursors (e.g., pyruvate and α-ketoglutarate) for amino acid biosynthesis [36]. Concurrently, sufficient glucose availability suppressed gluconeogenic demand, reducing the catabolic consumption of glucogenic amino acids (e.g., alanine and glutamine). This metabolic change creates a net accumulation of both essential (diet-derived) and non-essential (de novo-synthesized) amino acid pools.

Aminoacyl-tRNAs serve as critical molecular adapters that bridge genetic information with protein synthesis by accurately translating mRNA codons into corresponding amino acids. Our metabolomic analysis identified aminoacyl-tRNA biosynthesis as the most prominently altered pathway in NBW-treated rats, with 25.4% (17/67) of pathway-associated amino acids increased. This marked upregulation suggests enhanced translational capacity in hepatocytes, potentially facilitating increased protein production.

Nitrogen serves as a fundamental building block for multiple classes of biomolecules, ranging from amino acids and nucleotides to polyamines and hexosamines [37]. In rapidly proliferating cells, amino acids account for most of the total cellular mass [38], highlighting their central role in cell growth and maintenance. Particularly, glutamine and glutamate are noteworthy; these function as primary nitrogen carriers and metabolic regulators in cellular nitrogen homeostasis. Our experimental data revealed significant elevations in four amino acids (glutamic acid, glutamine, histidine, and glycine), representing 44.4% of the compounds in the nitrogen metabolism pathway. This coordinated upregulation strongly indicates enhanced nitrogen retention and utilization efficiency in the livers of NBW-fed rats, creating favorable conditions for the accelerated biosynthesis of nitrogen-containing macromolecules.

Our metabolomic analysis revealed significant alterations in the alanine-aspartate-glutamate metabolism pathway and phenylalanine-tyrosine-tryptophan biosynthesis pathway. Asparagine, a non-essential amino acid, serves as a metabolic hub for producing the glucose, proteins, lipids and nucleotides that sustain cellular viability [39]. The excitatory neurotransmitters, glutamate and aspartate, maintain a delicate equilibrium with inhibitory GABA, where an imbalance may disrupt neural signaling and impair cognitive-motor functions [40]. Particularly, phenylalanine and tyrosine initiate dopamine biosynthesis—the precursor for noradrenaline and adrenaline [41]. The production of these catecholamines directly correlates with cerebral concentrations of their aromatic amino acid precursors (tryptophan, phenylalanine, tyrosine), which are dependent on systemic availability [42]. In summary, the consumption of NBW may affect the metabolism of some neurotransmitters; this needs to be further explored.

Metabolomic profiling revealed significant alterations in three metabolic pathways: histidine metabolism; arginine-proline metabolism; and glycine-serine-threonine metabolism. L-histidine, an indispensable amino acid, serves as a multifunctional regulator in proton buffering, metal ion chelation, reactive species scavenging, erythrocyte production, and histamine-mediated neurotransmission [43]. Proline emerges as a dual-function metabolite, being both the fundamental building block for collagen formation [44] and a critical modulator of cellular processes including protein translation, oxidative stress response, differentiation programming, and neurodevelopment [45]. Glycine and serine, although classified as non-essential amino acids, constitute vital circulating nutrients that fuel the anabolic production of proteins, nucleotides, and lipids [46]. These amino acids particularly contribute to one-carbon metabolism—a central metabolic network that generates biosynthetic precursors, maintains cellular redox equilibrium, and supplies methyl groups for methylation reactions [47]. The coordinated modulation of these pathways suggests that NBW may function as a redox homeostasis modulator through its systemic metabolic effects.

Metabolic profiling revealed marked upregulation of three key intermediates in the valine-leucine-isoleucine biosynthesis pathway. These branched-chain amino acids (BCAAs)—leucine, valine and isoleucine—collectively represent the most prevalent proteinogenic amino acids with distinctive aliphatic side chain structures [48]. Functionally, BCAAs serve dual metabolic roles, which are fundamental substrates for protein biosynthesis and critical metabolic precursors for sterol synthesis, ketogenesis, and gluconeogenesis [49]. Emerging evidence highlights their systemic regulatory functions in energy metabolism modulation, nutritional metabolism, intestinal well-being, and immune response regulation [50].

Consequently, the consumption of NBW boosts amino acid levels, which are crucial for protein synthesis, and may help to maintain a positive nitrogen balance and redox homeostasis.

### 4.3. Fatty Acids Metabolism

Our analysis revealed marked hepatic elevation of seven fatty acid species [methylsuccinic acid, ricinoleic acid, 10z-heptadecenoic acid, alpha-linolenic acid, eicosapentaenoic acid (EPA), arachidonic acid, and docosahexaenoic acid (DHA)], alongside seven short-chain fatty acids (acetic acid, 3-hydroxyisovaleric acid, butyric acid, caproic acid, ethylmethylacetic acid, isovaleric acid, and valeric acid) in NBW-treated rats.

Hepatic concentrations of three physiologically critical omega-3 polyunsaturated fatty acids (*n*-3 PUFAs)—DHA (22:6*n*-3), EPA (20:5*n*-3), and α-linolenic acid (ALA, 18:3*n*-3)—were markedly increased in NBW-administered rats. Substantial evidence indicates that these *n*-3 PUFAs play pivotal roles in neurodevelopment and visual acuity during gestation, while conferring protective effects against multiple chronic pathologies including cardiovascular disorders, metabolic diseases, neurodegenerative conditions, and malignancies [51]. Concurrently, we observed arachidonic acid (AA, 20:4*n*-6), an indispensable *n*-6 series fatty acid that constitutes the structural components of cellular membranes and serves as the primary substrate for eicosanoid biosynthesis (such as encompassing prostaglandins and leukotrienes) [52]. Notably, mammals lack endogenous synthesis pathways for both *n*-3 and *n*-6 fatty acids [51], suggesting that the observed elevation likely reflects enhanced intestinal uptake efficiency mediated by NBW intervention.

SCFAs, defined as saturated aliphatic acids containing 1–6 carbon atoms, are predominantly represented by acetate, propionate, and butyrate in the intestinal lumen. These microbial metabolites exert pleiotropic effects on host physiology through the modulation of immune responses, programmed cell-death pathways, inflammatory cascades, and lipid homeostasis [53]. Clinically, SCFAs demonstrate therapeutic potential in mitigating multiple chronic conditions, including metabolic disorders, cardiovascular diseases, and gastrointestinal pathologies [53]. The biogenesis of SCFAs primarily depends on the anaerobic fermentation of complex carbohydrates by gut microbiota [54]. Notably, while all experimental animals received identical feed regimens, NBW consumption potentially enhanced SCFAs bioavailability through two synergistic mechanisms: remodeling gut microbial ecology by reducing the *Firmicutes/Bacteroidetes* ratio; and promoting probiotic proliferation (e.g., *Akkermansia muciniphila* and *Dubosiella* spp.), as evidenced in recent intervention studies on bicarbonate-rich mineral water [55].

This study has several limitations. First, our observations were limited to the metabolic alterations in 10-month-old female rats. Second, the duration of the experiment was restricted to three months, highlighting the need for further investigation into the metabolic alterations in both male and female rats over an extended period. Third, the molecular mechanisms underlying these metabolic alterations were not explored. Future research could focus on elucidating the effects of NBW consumption on gut microbiota dynamics and hepatic metabolomic regulation.

## 5. Conclusions

This study implemented a metabolomics strategy to systematically characterize the metabolic profile alterations induced by NBW consumption. Hepatic metabolic analysis revealed significant perturbations in 69 metabolites (67 upregulated and 2 downregulated) and 10 associated pathways in NBW-treated rats. The observed metabolic reprogramming demonstrates three key physiological impacts: (1) the establishment of positive nitrogen equilibrium; (2) upregulation of several polyunsaturated fatty acids and short-chain fatty acids; and (3) improvement in calcium homeostasis. These coordinated metabolic adaptations provide mechanistic insights into the documented health benefits of NBW intervention.

## Figures and Tables

**Table 1:** Drinking water composition.

Index	pH	TDS mg/L	TH mg/L	HCO_3_^−^ mg/L	SO_4_^2−^ mg/L	Cl^−^ mg/L	Boron mg/L	Ca^2+^ mg/L	Mg^2+^ mg/L	K^+^ mg/L	Na^+^ mg/L	H_2_SiO_3_ mg/L	COD_Mn_ mg/L	PRAL mEq/L
PW	6.33	3.63	0.43	0	0	0	0	0.17	0.08	0.04	0.33	0	0.50	−0.02
NBW	9.10	508.20	12.51	319.10	1.63	1.63	0.97	4.23	0.47	0.81	136.50	15.14	0.79	−5.64

PW, Purified water; NBW, Natural bicarbonate water; TDS, Total dissolved solids; TH, Total hardness; HCO_3_^−^, Bicarbonate; SO_4_^2−^, Sulfate; Cl^−^, Chloride; Ca, Calcium; Mg, Magnesium; K, Potassium; Na, Sodium; H_2_SiO_3_, Silicic acid; COD, Chemical oxygen demand; PRAL, potential renal acid load. PRAL (mEq/L) = [0.027 × Cl^−^ (mg/L) + 0.0146 × SO_4_^2−^ (mg/L)] − [0.021 × K^+^ (mg/L) + 0.0263 × Mg^2+^ (mg/L) +0.013 × Ca^2+^ (mg/L) + 0.0413 × Na^+^ (mg/L)]. The PRAL was calculated according to a previous study [7].

**Table 2:** Mineral concentrations in rat serum and urine (Mean ± SD).

Index		PW	NBW	*p* Value
Serum mineral	Sodium, mmol/L	135.84 ± 1.36	135.24 ± 2.51	0.51
	Potassium, mmol/L	9.71 ± 1.34	9.75 ± 1.56	0.96
	Calcium, mmol/L	2.69 ± 0.14	2.62 ± 0.12	0.28
	Magnesium, mmol/L	1.21 ± 0.09	1.22 ± 0.15	0.88
Urinary mineral	Sodium/Cr, mg/mg creatinine	2.74 ± 0.84	2.88 ± 0.75	0.68
	Potassium/Cr, mg/mg creatinine	6.96 ± 1.72	6.86 ± 1.54	0.88
	Calcium/Cr, mg/mg creatinine	0.42 ± 0.20	0.27 ± 0.17	0.08
	Magnesium/Cr, mg/mg creatinine	0.11 ± 0.12	0.12 ± 0.16	0.98

PW, Purified water; NBW, Natural bicarbonate water. Values are mean ± SD, *n* = 10 rats/group.

**Table 3:** Serum and urine biochemical profiles in rats (Mean ± SD).

	Index	PW	NBW	*p* Value
Serum lipid	Triglyceride, mmol/L	3.37 ± 2.42	4.88 ± 2.81	0.74
	Total cholesterol, mmol/L	3.00 ± 0.72	2.90 ± 0.65	0.21
	High-density lipoprotein, mmol/L	0.68 ± 0.11	0.63 ± 0.16	0.45
	Low-density lipoprotein, mmol/L	0.37 ± 0.12	0.35 ± 0.09	0.66
	AI	3.43 ± 0.95	3.74 ± 1.23	0.54
Hepatic enzyme biomarkers	Alanine aminotransferase, IU/L	81.69 ± 26.67	114.05 ± 73.22	0.21
	Aspartate aminotransferase, IU/L	185.41 ± 99.85	239.37 ± 151.13	0.35
	Direct bilirubin, μmol/L	0.58 ± 0.11	0.50 ± 0.14	0.14
	alkaline phosphatase, U/L	72.01 ± 30.45	79.69 ± 41.16	0.64
Renal function index	Urea, mmol/L	7.18 ± 1.90	7.53 ± 1.25	0.64
	Creatinine, μmol/L	42.22 ± 6.72	38.16 ± 5.17	0.15
	Uric acid, μmol/L	100.66 ± 40.93	90.19 ± 16.54	0.46
	Cystatin C, mg/L	0.07 ± 0.03	0.05 ± 0.03	0.54
	Retinol-binding protein, mg/L	2.80 ± 0.63	2.70 ± 0.67	0.74
Serum protein	Total protein, g/L	77.36 ± 4.81	74.89 ± 5.31	0.29
	Albumin, g/L	35.22 ± 2.84	33.63 ± 2.85	0.23
	Globulin, g/L	42.14 ± 3.62	41.26 ± 3.08	0.57
	Albumin/Globulin	0.84 ± 0.09	0.82 ± 0.06	0.46
	Prealbumin, mg/L	2.80 ± 0.92	2.10 ± 1.10	0.14
Urinary parameter	pH	7.77 ± 0.82	7.77 ± 0.85	1.00
	Urea/Cr, mmol/μmol creatinine	53.97 ± 19.57	49.37 ± 9.68	0.49
	Uric acid/Cr, μmol/μmol creatinine	0.15 ± 0.05	0.14 ± 0.05	0.59
	Creatinine, μmol/L	360.79 ± 94.99	268.02 ± 103.02	0.04

PW stands for purified water, and NBW for natural bicarbonate water. AI, Atherosclerotic index = (TCH − HDL)/HDL; TCH, Total cholesterol; HDL, High-density lipoprotein. Values are mean ± SD, *n* = 10 rats/group.

**Table 4:** Differential metabolites in the rat livers.

	Metabolite	Class	HMDB	KEGG	*p*-Value	FDR	FC	VIP	Trend
1	Lysine	Amino Acids	HMDB0000182	C00047	1.60 × 10^−4^	2.09 × 10^−3^	1.45	1.78	↑
2	Histidine	Amino Acids	HMDB0000177	C00135	2.18 × 10^−5^	4.27 × 10^−4^	1.49	1.91	↑
3	Ornithine	Amino Acids	HMDB0000214	C00077	8.39 × 10^−4^	6.49 × 10^−3^	1.48	1.65	↑
4	Glutamine	Amino Acids	HMDB0000641	C00064	7.64 × 10^−3^	3.07 × 10^−2^	1.30	1.32	↑
5	Glutamic acid	Amino Acids	HMDB0000148	C00025	6.95 × 10^−6^	2.66 × 10^−4^	1.51	1.88	↑
6	Cystine	Amino Acids	HMDB0000192	C00491	9.86 × 10^−3^	3.89 × 10^−2^	1.49	1.24	↑
7	Sarcosine	Amino Acids	HMDB0000271	C00213	3.89 × 10^−3^	2.03 × 10^−2^	2.00	1.37	↑
8	β-Alanine	Amino Acids	HMDB0000056	C00099	4.87 × 10^−4^	4.43 × 10^−3^	2.12	1.82	↑
9	Alanine	Amino Acids	HMDB0000161	C00041	1.08 × 10^−5^	2.66 × 10^−4^	1.58	1.91	↑
10	Dimethylglycine	Amino Acids	HMDB0000092	C01026	4.01 × 10^−2^	1.15 × 10^−1^	1.42	1.07	↑
11	γ-aminobutyric acid	Amino Acids	HMDB0000112	C00334	2.88 × 10^−3^	1.57 × 10^−2^	1.68	1.51	↑
12	Serine	Amino Acids	HMDB0000187	C00065	1.23 × 10^−3^	8.89 × 10^−3^	1.40	1.69	↑
13	Methylcysteine	Amino Acids	HMDB0002108	NA	2.13 × 10^−4^	2.35 × 10^−3^	1.81	1.76	↑
14	2-Phenylglycine	Amino Acids	HMDB0002210	NA	6.17 × 10^−3^	2.73 × 10^−2^	1.81	1.33	↑
15	Tyrosine	Amino Acids	HMDB0000158	C00082	2.25 × 10^−5^	4.27 × 10^−4^	1.57	1.88	↑
16	Asparagine	Amino Acids	HMDB0000168	C00152	9.43 × 10^−5^	1.31 × 10^−3^	1.65	1.82	↑
17	Phenylalanine	Amino Acids	HMDB0000159	C00079	2.09 × 10^−3^	1.25 × 10^−2^	1.20	1.22	↑
18	Kynurenine	Amino Acids	HMDB0000684	C00328	2.14 × 10^−2^	7.22 × 10^−2^	2.08	1.28	↑
19	Aspartic acid	Amino Acids	HMDB0000191	C00049	9.50 × 10^−4^	7.09 × 10^−3^	1.33	1.58	↑
20	Glycine	Amino Acids	HMDB0000123	C00037	7.00 × 10^−5^	1.05 × 10^−3^	1.37	1.72	↑
21	Proline	Amino Acids	HMDB0000162	C00148	1.14 × 10^−6^	2.37 × 10^−4^	1.87	2.04	↑
22	Acetylglycine	Amino Acids	HMDB0000532	NA	1.08 × 10^−5^	2.66 × 10^−4^	1.37	1.87	↑
23	Pipecolic acid	Amino Acids	HMDB0000716	C00408	5.42 × 10^−3^	2.46 × 10^−2^	1.44	1.39	↑
24	N-Acetylserine	Amino Acids	HMDB0002931	NA	3.37 × 10^−6^	2.43 × 10^−4^	1.82	1.95	↑
25	N-Acetylglutamine	Amino Acids	HMDB0006029	NA	1.69 × 10^−2^	6.09 × 10^−2^	1.21	1.16	↑
26	Valine	Amino Acids	HMDB0000883	C00183	1.35 × 10^−3^	9.11 × 10^−3^	1.36	1.55	↑
27	Pyroglutamic acid	Amino Acids	HMDB0000267	C01879	3.49 × 10^−6^	2.43 × 10^−4^	1.73	1.93	↑
28	Methionine	Amino Acids	HMDB0000696	C00073	6.68 × 10^−4^	5.59 × 10^−3^	1.42	1.66	↑
29	Isoleucine	Amino Acids	HMDB0000172	C00407	1.51 × 10^−3^	9.25 × 10^−3^	1.42	1.30	↑
30	Leucine	Amino Acids	HMDB0000687	C00123	2.12 × 10^−4^	2.35 × 10^−3^	1.37	1.74	↑
31	Tryptophan	Amino Acids	HMDB0000929	C00078	4.33 × 10^−5^	7.54 × 10^−4^	1.33	1.67	↑
32	1-Methylhistidine	Amino Acids	HMDB0000001	C01152	3.63 × 10^−4^	3.45 × 10^−3^	1.45	1.56	↑
33	α-Aminobutyric acid	Amino Acids	HMDB0000452	C02356	4.80 × 10^−3^	2.43 × 10^−2^	1.57	1.45	↑
34	5-Aminolevulinic acid	Amino Acids	HMDB0001149	C00430	5.14 × 10^−4^	4.48 × 10^−3^	1.59	1.68	↑
35	ortho-Hydroxyphenylacetic acid	Benzenoids	HMDB0000669	C05852	2.10 × 10^−2^	7.19 × 10^−2^	0.61	1.15	↓
36	Gluconolactone	Carbohydrates	HMDB0000150	C00198	2.39 × 10^−2^	7.93 × 10^−2^	1.48	1.16	↑
37	N-Acetylneuraminic acid	Carbohydrates	HMDB0000230	C00270	5.84 × 10^−5^	9.39 × 10^−4^	1.78	1.86	↑
38	Glucose	Carbohydrates	HMDB0000122	C00221	8.64 × 10^−6^	2.66 × 10^−4^	1.62	1.93	↑
39	Lactulose	Carbohydrates	HMDB0000740	C07064	1.15 × 10^−5^	2.66 × 10^−4^	1.87	1.95	↑
40	Maltose/Lactose	Carbohydrates	NA	NA	1.08 × 10^−5^	2.66 × 10^−4^	2.06	2.00	↑
41	Maltotriose	Carbohydrates	HMDB0001262	C01835	2.93 × 10^−3^	1.57 × 10^−2^	1.50	1.51	↑
42	Rhamnose	Carbohydrates	HMDB0000849	C00507	3.68 × 10^−2^	1.07 × 10^−1^	1.50	1.03	↑
43	Fructose	Carbohydrates	HMDB0000660	C02336	5.20 × 10^−3^	2.43 × 10^−2^	1.66	1.28	↑
44	N-Acetyl-D-glucosamine	Carbohydrates	HMDB0000215	C00140	7.25 × 10^−4^	5.83 × 10^−3^	1.79	1.76	↑
45	Fructose 6-phosphate	Carbohydrates	HMDB0000124	C00085	4.87 × 10^−2^	1.30 × 10^−1^	1.35	1.17	↑
46	Ribonic acid	Carbohydrates	HMDB0000867	C01685	3.25 × 10^−4^	3.23 × 10^−3^	2.05	1.51	↑
47	Butyrylcarnitine	Carnitines	HMDB0002013	C02862	3.25 × 10^−2^	9.71 × 10^−2^	2.08	1.01	↑
48	3-Hydroxylisovalerylcarnitine	Carnitines	NA	NA	3.00 × 10^−2^	9.37 × 10^−2^	1.30	1.17	↑
49	Methylsuccinic acid	Fatty Acids	HMDB0001844	NA	4.33 × 10^−2^	1.17 × 10^−1^	1.39	1.32	↑
50	Ricinoleic acid	Fatty Acids	HMDB0034297	C08365	1.80 × 10^−2^	6.38 × 10^−2^	1.44	1.04	↑
51	10Z-Heptadecenoic acid	Fatty Acids	HMDB0060038	NA	1.85 × 10^−2^	6.46 × 10^−2^	1.87	1.11	↑
52	α-Linolenic acid	Fatty Acids	HMDB0001388	C06427	6.84 × 10^−3^	2.80 × 10^−2^	1.79	1.33	↑
53	Eicosapentaenoic acid	Fatty Acids	HMDB0001999	C06428	5.20 × 10^−3^	2.43 × 10^−2^	2.28	1.31	↑
54	Arachidonic acid	Fatty Acids	HMDB0001043	C00219	6.84 × 10^−3^	2.80 × 10^−2^	1.53	1.28	↑
55	Docosahexaenoic acid	Fatty Acids	HMDB0002183	C06429	2.88 × 10^−3^	1.57 × 10^−2^	1.60	1.40	↑
56	Glutaric acid	Organic Acids	HMDB0000661	C00489	5.20 × 10^−3^	2.43 × 10^−2^	1.35	1.53	↑
57	Aconitic acid	Organic Acids	HMDB0000072	C02341	3.05 × 10^−2^	9.37 × 10^−2^	0.89	1.23	↓
58	3-Hydroxybutyric acid	Organic Acids	HMDB0000357	C01089	1.51 × 10^−3^	9.25 × 10^−3^	1.43	1.36	↑
59	2-Hydroxybutyric acid	Organic Acids	HMDB0000008	C05984	1.51 × 10^−3^	9.25 × 10^−3^	1.54	1.49	↑
60	Acetoacetic acid	Organic Acids	HMDB0000060	C00164	1.58 × 10^−2^	5.80 × 10^−2^	1.52	1.17	↑
61	Glycylproline	Peptides	HMDB0000721	NA	5.23 × 10^−3^	2.43 × 10^−2^	1.60	1.40	↑
62	Homovanillic acid	Phenols	HMDB0000118	C05582	3.25 × 10^−4^	3.23 × 10^−3^	1.51	1.49	↑
63	Acetic acid	SCFAs	HMDB0000042	C00033	1.04 × 10^−2^	3.96 × 10^−2^	1.47	1.47	↑
64	3-Hydroxyisovaleric acid	SCFAs	HMDB0000754	NA	3.12 × 10^−2^	9.44 × 10^−2^	1.53	1.08	↑
65	Butyric acid	SCFAs	HMDB0000039	C00246	2.05 × 10^−4^	2.35 × 10^−3^	1.97	1.66	↑
66	Caproic acid	SCFAs	HMDB0000535	C01585	6.27 × 10^−3^	2.73 × 10^−2^	1.57	1.24	↑
67	Ethylmethylacetic acid	SCFAs	HMDB0002176	C18319	2.76 × 10^−3^	1.57 × 10^−2^	1.92	1.60	↑
68	Isovaleric acid	SCFAs	HMDB0000718	C08262	1.31 × 10^−3^	9.11 × 10^−3^	2.73	1.69	↑
69	Valeric acid	SCFAs	HMDB0000892	C00803	2.51 × 10^−2^	8.19 × 10^−2^	1.34	1.25	↑

VIP scores (threshold ≥ 1.0) were derived from OPLS-DA modeling, with *p*-values calculated using Student’s *t*-test or Mann–Whitney U-test based on data distribution characteristics. Arrows indicate metabolite concentration changes (↓ decreased, ↑ increased). Database abbreviations: HMDB (Human Metabolome Database) and KEGG (Kyoto Encyclopedia of Genes and Genomes). Sample size: *n* = 10 rats/group.

**Table 5:** Significantly altered metabolic pathways in rat liver.

	Total in Pathway	Hits	Raw *p*	FDR	Impact	Enriched Compounds	KEGG Link
Aminoacyl-tRNA biosynthesis	67	17	1.45 × 10^−10^	1.18 × 10^−8^	0.37	Asparagine, Histidine, Phenylalanine, Glutamine, Glycine, Aspartic acid, Serine, Methionine, Valine, Alanine, Lysine, Isoleucine, Leucine, Tryptophan, Tyrosine, Proline, Glutamic acid	http://www.genome.jp/kegg-bin/show_pathway?rno00970/C00152%09red/C00135%09red/C00079%09red/C00064%09red/C00037%09red/C00049%09red/C00065%09red/C00073%09red/C00183%09red/C00041%09red/C00047%09red/C00407%09red/C00123%09red/C00078%09red/C00082%09red/C00148%09red/C00025%09red (Accessed on 27 May 2025)
Alanine, aspartate and glutamate metabolism	24	6	2.64 × 10^−4^	7.19 × 10^−3^	0.64	Aspartic acid, Alanine, Glutamic acid, GABA, Glutamine, Asparagine	http://www.genome.jp/kegg-bin/show_pathway?rno00250/C00049%09red/C00041%09red/C00025%09red/C00334%09red/C00064%09red/C00152%09red (Accessed on 27 May 2025)
Nitrogen metabolism	9	4	2.66 × 10^−4^	7.19 × 10^−3^	0.44	Glutamic acid, Glutamine, Histidine, Glycine	http://www.genome.jp/kegg-bin/show_pathway?rno00910/C00025%09red/C00064%09red/C00135%09red/C00037%09red (Accessed on 27 May 2025)
Butanoate metabolism	20	5	9.04 × 10^−4^	1.83 × 10^−2^	0.28	3-Hydroxybutyric acid, Acetoacetic acid, GABA, Glutamic acid, Butyric acid	http://www.genome.jp/kegg-bin/show_pathway?rno00650/C01089%09red/C00164%09red/C00334%09red/C00025%09red/C00246%09red (Accessed on 27 May 2025)
Histidine metabolism	15	4	2.4 × 10^−3^	3.89 × 10^−2^	0.31	Glutamic acid, Histidine, Aspartic acid, 1-Methylhistidine	http://www.genome.jp/kegg-bin/show_pathway?rno00340/C00025%09red/C00135%09red/C00049%09red/C01152%09red (Accessed on 27 May 2025)
Phenylalanine metabolism	9	3	4.50 × 10^−3^	6.07 × 10^−2^	0.44	Phenylalanine, ortho-Hydroxyphenylacetic acid, Tyrosine	http://www.genome.jp/kegg-bin/show_pathway?rno00360/C00079%09red/C05852%09red/C00082%09DeepSkyBlue (Accessed on 27 May 2025)
Arginine and proline metabolism	44	6	7.35 × 10^−3^	7.49 × 10^−2^	0.22	Glutamine, Ornithine, Aspartic acid, Glutamic acid, Proline, GABA	http://www.genome.jp/kegg-bin/show_pathway?rno00330/C00064%09red/C00077%09red/C00049%09red/C00025%09red/C00148%09red/C00334%09red (Accessed on 27 May 2025)
Glycine, serine and threonine metabolism	32	5	8.07 × 10^−3^	7.49 × 10^−2^	0.43	Serine, Dimethylglycine, Glycine, Sarcosine, 5-Aminolevulinic acid	http://www.genome.jp/kegg-bin/show_pathway?rno00260/C00065%09red/C01026%09red/C00037%09red/C00213%09red/C00430%09red (Accessed on 27 May 2025)
Valine, leucine and isoleucine biosynthesis	11	3	8.34 × 10^−3^	7.49 × 10^−2^	0.57	Leucine, Valine, Isoleucine	http://www.genome.jp/kegg-bin/show_pathway?rno00290/C00123%09red/C00183%09red/C00407%09red (Accessed on 27 May 2025)
Phenylalanine, tyrosine and tryptophan biosynthesis	4	2	9.25 × 10^−3^	7.49 × 10^−2^	0.75	Phenylalanine, Tyrosine	http://www.genome.jp/kegg-bin/show_pathway?rno00400/C00079%09red/C00082%09red (Accessed on 27 May 2025)

Hits: Count of differential metabolites contained in this pathway; Raw *p*, *p*-value of pathway enrichment analysis; FDR: False discovery rate; Impact: Pathway topological impact score.

## Data Availability

The original contributions presented in the study are included in the article/Supplementary Materials; further inquiries can be directed to the corresponding authors.

## Supplementary Materials

The following supporting information can be downloaded at: https://www.mdpi.com/article/10.3390/nu17111875/s1, Supplementary Materials and Methods Section: Sample preparation and derivatization protocols for metabolomics analysis; Instrument settings of the UPLC-MS/MS analysis; Table S1: Food composition. Figure S1: Body weight, water intake and food intake.
