# Supplementary material for: Natural Bicarbonate Water Might Enhance Nitrogen Balance and Lipid Metabolism and Improve Calcium Balance: A Full Quantitative Targeted Metabolomics Study in Rats
Source: Nutrients. 2025 May 30;17(11):1875. doi: 10.3390/nu17111875 (PMC12157924; doi:10.3390/nu17111875)

Supplementary data

Natural Bicarbonate Water Might Enhance Nitrogen Balance and Lipid Metabolism and Improve Calcium Balance: A Full Quantitative Targeted Metabolomics Study in Rats

Jiaohua Luo [1,†], Jia Wang [2,†], Zhiqun Qiu [1], Hui Zeng [1], Yao Tan [1], Yujing Huang [1,*] and Weiqun Shu [1,*]

[1]Department of Environmental Hygiene, College of Preventive Medicine, Army Medical University, Chongqing, 400038, China

[2]Department of Medical English, College of Basic Medicine, Army Medical University, Chongqing, China

*Correspondence: huangyujing@tmmu.edu.cn; weiqunshu@tmmu.edu.cn.

[†]These authors contributed equally to this work.

Table of contents

1. Supplementary materials and methods

1.1 Sample preparation and derivatization protocols

1.2 Instrument settings of the UPLC-MS/MS analysis

2. Supplementary table and figure

2.1 Supplementary Table S1. Composition of the feed

2.2 Supplementary Figure S1. Body weight, water intake and food intake

1. Supplementary materials and methods

1.1 Sample preparation and derivatization protocols

Each liver sample (~10mg) was mixed with 10 pre-chilled zirconium oxide beads and 20μL of deionized water. The sample was homogenated for 3 minutes and then mixed with 120μL of methanol containing internal standard. The sample was homogenated for another 3 minutes and then centrifuged at 18000g for 20 minutes. The supernatant was transferred to a 96-well plate. The following procedures were performed on an Eppendorf epMotion Workstation (Eppendorf Inc., Humburg, Germany). An aliquot of 20μL of freshly prepared derivative reagents was added to each well. The plate was sealed and incubated at 30°C for 60 minutes. Following derivatization, the sample was evaporated for 2h. The sample was reconstituted with 330μL of ice-cold 50% methanol solution. Then the plate was stored at -20°C for 20 minutes and centrifuged at 4000g for 30 minutes at 4°C. Supernatant (135μL) was transferred to a new 96-well plate with 10μL internal standards in each well. Derivatized stock standards were added to the left wells. Finally, the plate was sealed for LC-MS analysis.

1.2 **Instrument settings of the UPLC-MS/MS analysis**

An ultra-performance liquid chromatography coupled to tandem mass spectrometry (UPLC-MS/MS) system (ACQUITY UPLC-Xevo TQ-S, Waters Corp., Milford, MA, USA) was used. The instrument settings were as follows. The ultra-high performance liquid chromatography system was equipped with a BEH C18 1.7 μm VanGuard pre-column and a BEH C18 1.7 μm analytical column. The column temperature was maintained at 40 °C, with a flow rate of 0.4 mL/min. The sample tray temperature was set to 10 °C, and the sample volume was 5 μL. The mobile phase consisted of A (water containing 0.1% formic acid) and B (a mixture of acetonitrile and isopropanol in a 70:30 ratio). The gradient elution procedure was 0-1 min 5% B, 1-11 min 5% ~ 78% B, 11-13.5 min 78% ~ 95%B, 13.5-14 min 95% ~ 100% B, 14-16 min 100% B, 16-16.1 min 100% ~ 5% B, and 16.1-18 min 5% B.

Electrospray ionization mass spectrum (ESI-MS) was used. The capillary voltage was set to 1.5 kV in positive mode (ESI +) and 2.0 kV in negative mode (ESI-). The source temperature was maintained at 150 °C. The desolvation temperature was set to 550 °C, with a flow rate of 1000 L/h.

The instrument performance optimization and routine maintenance were performed every week.

2. Supplementary table and figure

2.1 Table S1. Composition of the feed (per Kg)

Gradient	Weight	Gradient	Weight
Crude protein	⩾200g	Vitamin C	-
Crude fat	⩾40g	Vitamin K	⩾5.0mg
Crude fiber	⩽50g	Vitamin B_1	⩾13mg
Crude ash	⩽80g	Vitamin B_2	⩾12mg
Moisture content	⩽100g	Vitamin B_6	⩾12mg
Calcium	10-18g	Vitamin B_{12}	⩾0.022mg
Phosphorus	6-12g	Niacin	⩾60mg
Lysine	⩾13.2g	Pantothenic acid	⩾24mg
Methionine + Cystine	⩾7.8g	Folate	⩾6.0mg
Arginine	⩾11g	Biotin	⩾0.2mg
Histidine	⩾5.5g	Choline	⩾1250mg
Tryptophan	⩾2.5g	Magnesium	⩾2.0g
Phenylalanine + Tyrosine	⩾13g	Potassium	5.0g
Threonine	⩾8.8g	Sodium	⩾2.0g
Leucine	⩾17.6g	Ferrum	⩾120mg
Isoleucine	⩾10.3g	Manganese	⩾75mg
Valine	⩾11.7g	Copper	⩾10mg
Vitamin A	⩾14000IU	Zinc	⩾30mg
Vitamin D	⩾1500IU	Iodine	⩾0.5mg
Vitamin E	⩾120IU	Selenium	0.1-0.2mg

The composition of the feed was strictly followed the standard of GB14924-2010 in China for experimental animal feed nutrition.

2.2 Supplementary Figure S1

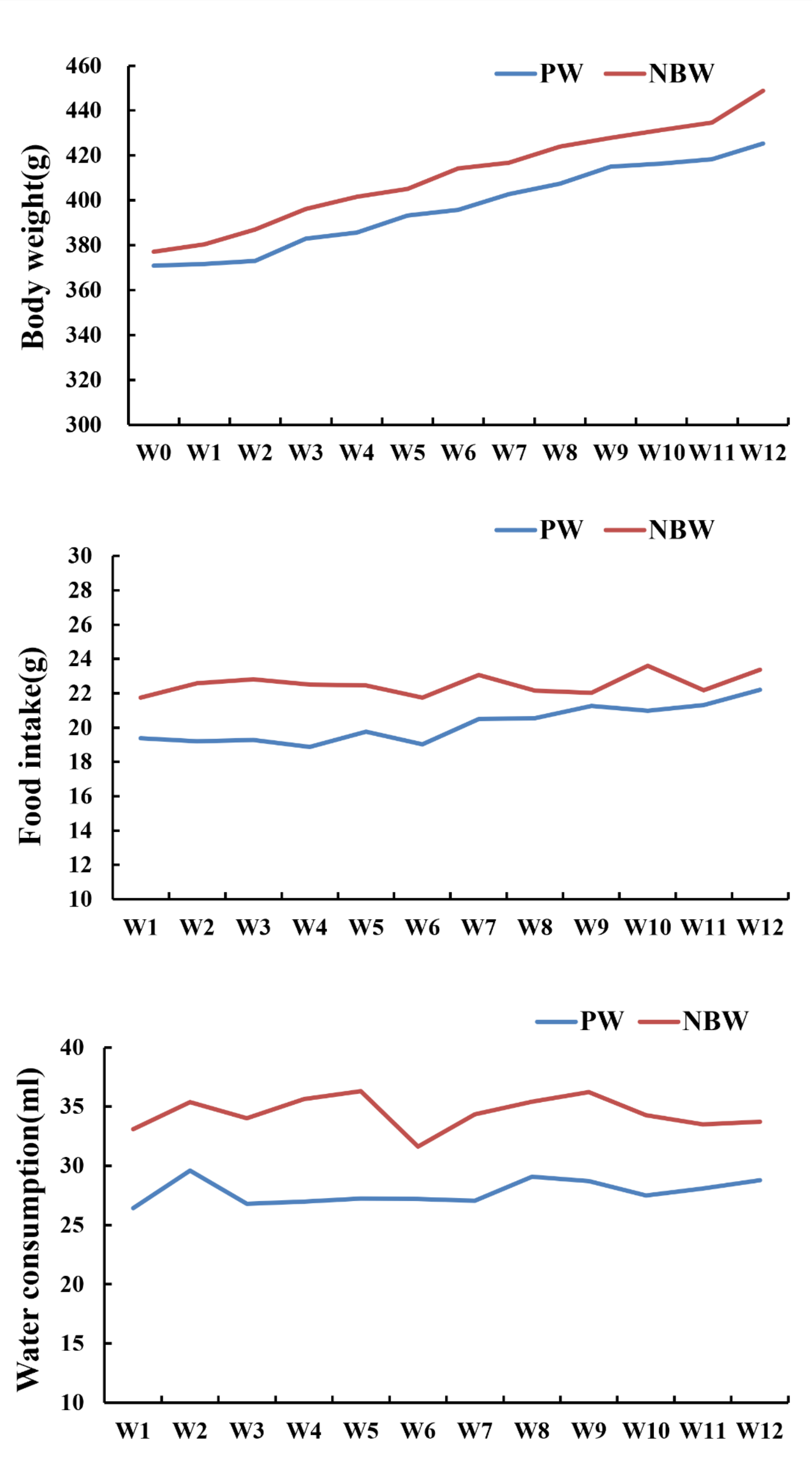

Supplement: Supplementary file 1 [file nutrients-17-01875-s001.zip › nutrients-3626521-supplementary.pdf]
